# Highly Sensitive Pressure Transducer for Measuring Arterial Pulse Wave Velocity Based on Giant Magneto-Impedance Sensors

**DOI:** 10.3390/s25103188

**Published:** 2025-05-19

**Authors:** Lizeth Stefanía Benavides Cabrera, Eduardo Costa da Silva, Elisabeth Costa Monteiro

**Affiliations:** 1 Department of Electrical Engineering, Pontifical Catholic University of Rio de Janeiro, Rio de Janeiro 22451-900, Brazil; sthepany_07@hotmail.com; 2 Samsung R&D Institute Brazil, Campinas, São Paulo 13097-104, Brazil; 3 Postgraduate Programme in Metrology, Pontifical Catholic University of Rio de Janeiro, Rio de Janeiro 22451-900, Brazil; beth@puc-rio.br

**Keywords:** giant magneto-impedance, impedance phase, GMI magnetometer, pressure transducer, arterial pulse wave, pulse wave velocity

## Abstract

**Highlights:**

A novel pressure transducer based on phase readings of GMI sensors enables non-contact, high-sensitivity measurement of arterial pulse waves.

The proposed system eliminates the need for mechanical amplification, improving spatial resolution and usability in hard-to-reach anatomical regions, thus providing a contactless method unaffected by optical factors such as ambient light intensity or skin melanin density.

**What are the main findings?**

- The developed GMI-based magnetometer successfully captures arterial pulse waveforms and estimates pulse wave velocity (PWV) with high sensitivity and resolution.
- A contactless approach using magnetic markers and phase-sensitive GMI sensors was tested in a proof-of-concept setup for simultaneous pulse wave acquisition at multiple arterial sites.

**What is the implication of the main finding?**

- This innovation provides a safe, non-invasive, and cost-effective alternative to conventional pressure transducers for cardiovascular monitoring, unaffected by influences associated with conventional measuring approaches caused by factors such as the angle and amplitude of the incident force of contact between the probe and the skin, the probe’s dimension, and optical aspects.
- The proposed system has the potential to enhance clinical diagnostics and expand access to cardiovascular assessment tools.

**Abstract:**

Pulse wave velocity (PWV) has been recognised as the gold standard for assessing arterial stiffness and a relevant indicator in diagnosing cardiovascular disease. Conventional approaches can be affected by factors such as the size of the probe, its positioning on the skin with the appropriate angle and magnitude of the incident force, or influenced by optical properties. Aiming at improving the assessment of PWV parameter, an important cardiovascular risk marker, the present study introduces a new arterial pulse wave measurement technique based on measurements of the impedance phase characteristics of giant magneto-impedance (GMI) sensors submitted to slight magnetic field variations caused by the displacement of a small magnetic marker placed on the patient’s skin, whose movement is coordinated by the local pressure wave. The proposed method eliminates the necessity of using probes with mechanical amplification, enhancing spatial resolution and usability in hard-to-reach anatomical regions through a contactless device unaffected by optical parameters. The obtained experimental results indicate the potential of the developed measurement system in measuring arterial pulse waveform and PWV.

## 1. Introduction

According to a World Health Organisation (WHO) report, Cardiovascular Disease (CVD) is the leading cause of death globally. Among these deaths, 85% were due to heart attack and stroke [1]. Recent studies have shown that changes in the mechanical properties of the artery wall can be used as a predictor of cardiovascular disease and as an indicator of mortality risk. So, special attention has been focused on pulse wave velocity (PWV) as a reliable and straightforward non-invasive tool to improve detection and risk stratification for CVD. The PWV measurement is the gold standard for evaluating arterial stiffness [2,3,4]. Many clinical studies have shown the association between PWV and atherosclerosis [5,6], with diabetes [6,7], and renal failure [6,8,9,10]. More recent studies have also linked higher pulse wave velocity levels to an increased incidence of cerebral microbleeds [11].

While intra-arterial measurement of the blood pressure waveform serves as the reference for determining systolic, diastolic, and mean arterial pressure, its invasive nature prevents it from being used in routine inspections. The associated risks of invasive approaches for assessing the physiological quantity have prompted the development of non-invasive alternatives. Multiple techniques enabling continuous recording of arterial waveforms have emerged in the pursuit of integrating these devices into clinical practice. Conventionally, arterial pressure transducers are based on piezoresistive, piezoelectric, capacitive, or optical sensors. These devices require skin contact and are influenced by external factors such as the contact force between the sensor probe and the skin, ambient light levels [12], and melanin concentration [13]. The main challenge in developing arterial pulse wave pressure transducers for clinical practice is to conceive low-cost, non-invasive solutions that provide good spatial resolution. Ideally, these solutions should not require direct contact with the skin and should be unaffected by ambient light intensity or skin melanin density.

Recent research has shown that high-sensitivity pressure transducers can be implemented using magnetic sensors based on giant magneto-impedance (GMI) [14]**.** GMI magnetometers are one of the most recent families of magnetic transducers. They use sensors based on the Giant Magneto-impedance effect as an intermediary block in the transduction process. The GMI effect is characterised by a considerable change in a ferromagnetic conductor’s impedance (magnitude and phase) upon applying an external magnetic field. This effect results from the skin depth dependency on the magnetic permeability, which varies not only with the external magnetic field applied to the sample but also with the amplitude and frequency of the current passing through it [15]. Further research indicated that phase-based GMI transducers are much more sensitive than the usual magnitude-based GMI transducers, increasing the sensitivity by at least 100 times [14,16].

**Figure 1:**
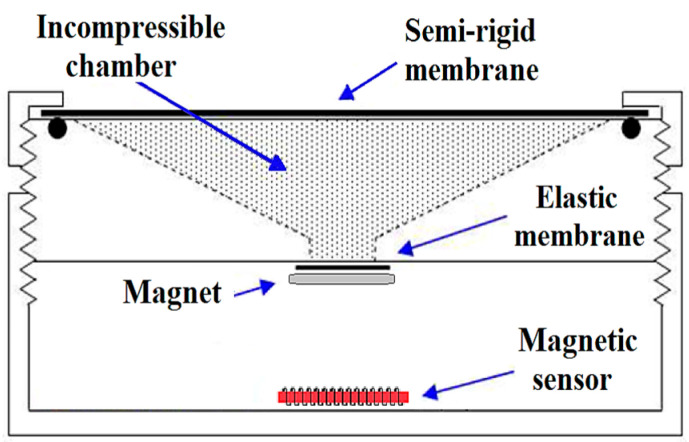
Configuration of the pressure transducer using a GMI sensor and an incompressible chamber [18].

So, aiming to take advantage of the highly sensitive GMI sensors in previous works [17,18], we developed a pressure transducer using a GMI magnetometer as part of the transduction chain of pressure into an output electric voltage. Figure 1 shows the configuration of the previous prototype, which includes an incompressible chamber for mechanical amplification.

In the pressure transducer shown in Figure 1, the incompressible chamber transmits a pressure variation applied to the semi-rigid membrane and displaces the elastic layer. This displacement moves the magnet toward the GMI magnetic sensor, which is placed at the base of the mechanical structure, generating variations in the magnetic field. The magnetic sensor is placed inside a solenoid that guarantees a proper biasing magnetic field for the sensor [18].

The diameter of the semi-rigid membrane employed in the prototype, presented in Figure 1, was equal to 3.2 cm. For measurement purposes, the transducer’s membrane is placed in direct contact with the skin at the appropriate anatomical location for the arterial pulse wave assessment. However, two aspects are relevant to the measurement quality. The first is the intensity of mechanical pressure applied to the skin when positioning the transducer probe at the appropriate site overlying the artery to be evaluated. Excessive force applied by the operator may pose risks to arterial flow or stimulate the carotid sinus in cases of hypersensitivity [19]; on the other hand, insufficient mechanical pressure may make the proper recording of the pulse waves difficult. The second important aspect to consider is the spatial resolution of pressure transducers. These devices contact the skin with a few centimeters in diameter membranes, limiting access to small anatomical regions that are more challenging to reach due to the local structures adjacent to the artery [20].

The present work aims to overcome difficulties associated with applying the pressure transducer apparatus to the skin, especially in regions that are difficult to reach. This paper presents a new, highly sensitive GMI magnetometer based on the impedance phase characteristics of the GMI sensor, developed for use with a small magnetic marker placed on the patient’s skin to record the arterial pulse waveform. The system does not require mechanical amplification structures, such as the one proposed in [18], allowing for contactless measurements unaffected by optical factors, including ambient light intensity and skin melanin density**.** This new diagnostic measuring instrument aims to address the essential aspects considered in metrology applied to the health sector, such as innocuity, non-invasiveness, accuracy, precision, portability, and low operation and production costs [21,22].

## 2. Arterial Pulse Wave Velocity

In the early 1900s, Crighton Bramwell first introduced the concept of pulse wave velocity (PWV) [23], which consists of the velocity of the pressure wave generated by ventricular ejection as it travels from the heart to the arterial vessels, being affected by the viscoelastic properties of the arteries. For example, increased stiffness of the arterial walls increases the propagation velocity of the pulse wave. Other properties, such as arterial wall thickness and cavity diameter [23], also significantly impact PWV. It is important to note that pulse wave velocity differs from blood flow velocity, which is the speed at which red blood cells and other elements move through the arterial system. Blood flow velocity varies significantly during the cardiac cycle, typically by a few cm/s, while pulse wave velocity is about a few m/s, ranging from 4 m/s to 20 m/s [24].

The conventional method used to measure PWV along an arterial segment *AB*, with a known length, consists of measuring the time interval (*dT*) that a specific pulse wave takes to travel from point *A* to point *B* within the analysed *AB* interval [24].

Subsequently, knowing the time *dT* that the pulse wave takes to travel the arterial segment *AB*, the velocity can be estimated using:

(1) $$VOP=\frac{AB}{dT}$$

**Figure 2:**
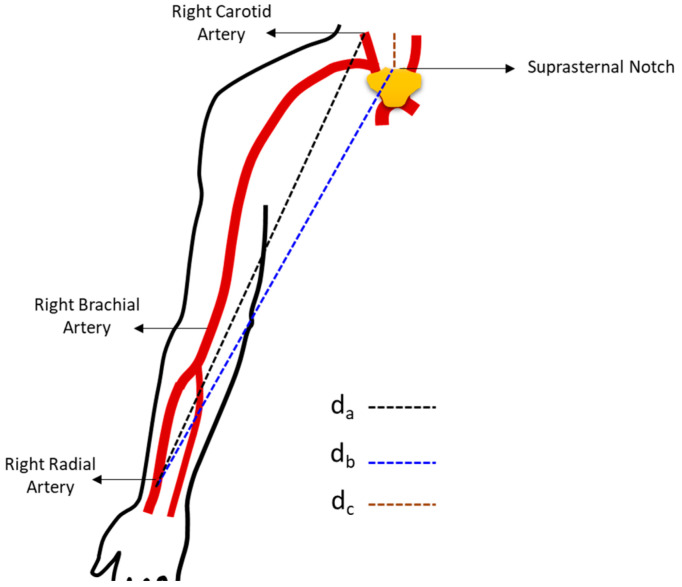
The main methods for assessing the carotid-radial distance in PWV evaluation: “direct” measurement of the carotid-radial distance, using distance (*d_a_*); “subtractive” method for measuring the carotid-radial distance, using lengths (*d_b_*) and (*d_c_*) [2].

However, pulse wave velocity values depend on the algorithm used to calculate the time *dT* and the technique for measuring the distance between the two recording sites (*AB*). The calculation of propagation time, also known as delay time, can be performed using various methods, with the ‘foot to foot’ method commonly employed. In this case, the start point of each pulse wave period serves as the reference point for calculating the time delay between two recordings. The delay time can be determined through a single measurement, using two transducers for simultaneous measurement at two points in the arterial tree (Figure 2) [2].

On the other hand, the pulse wave velocity values are also strongly dependent on the measurement of the length of the arterial segment *AB*. There are different ways to measure the distance, with the most commonly employed method being the direct measurement of the distance between the two measurement points (Figure 2), distance *d_a_*. However, this method leads to a systematic overestimation of PWV by approximately 30% [25]. Therefore, recent studies have shown that the subtractive process is the most appropriate technique for measuring this distance, where the sternal notch is taken as the reference point, and the distance between the carotid pulse measurement site and this reference point is determined [2], distance *d_b_* (Figure 2). Then, this value is subtracted from the distance between the sternal notch and the second measurement site (Figure 2), distance *d_c_*. In the example presented in Figure 2, the second measurement site corresponds to the radial artery.

The measurement of pulse wave velocity is a non-invasive and easily implementable tool that enables the evaluation of arterial stiffness and provides information about the mechanical properties of the arterial tree [26]. Non-invasive pulse wave velocity measurement is the gold standard for assessing arterial stiffness. However, to properly interpret arterial stiffness, it should be noted that it is influenced by several factors, including mechanical alterations of the arterial wall, individual genetic factors, sex, diseases, and age [3,27,28,29].

Pulse wave velocity (PWV) is a biomarker that has gained attention in clinical research [30,31]. Epidemiological and clinical studies have demonstrated that increased aortic stiffness, determined by measuring PWV, predicts cardiovascular risk in the general population. Furthermore, given the close interaction between arterial stiffness and atherosclerosis, PWV measurement can help diagnose individuals at high risk of developing this disease [5,6,29]. Significant increases in PWV have also been associated with diabetes [7] and renal insufficiency [6,8,9,10]. Therefore, measuring pulse waves with high-sensitivity, low-cost devices can benefit everyday clinical practice by monitoring treatment and preventing cardiac disorders.

In 2010, PWV was defined as the non-invasive reference standard for arterial stiffness measurement, and reference values were established [3]. The initial recommendation was to consider carotid-femoral PWVs exceeding 12 m/s as abnormal. However, subsequent studies conducted by the Working Group on Vascular Structure and Function of the European Society of Hypertension demonstrated that the standard value of 10 m/s is more suitable for the general population [32]. It is essential to be aware of the limitations of using this reference value, as PWV is influenced by various parameters (age, sex, weight, height, among others). Therefore, it is emphasized that this may not always be the most effective way to predict risk when dealing with highly diverse sample populations [29,32,33].

Contemporary cardiovascular (CV) monitoring trends are moving away from more invasive methods like arterial applanation tonometry, oscillometry, and plethysmography that require physical contact with the patient and involve compressing the artery throughout the cardiac cycle. These current measurement techniques are further complicated by the Bernoulli effect, which distorts the shape of the pulse curve itself [34,35]. Consequently, the existing devices face limitations associated with their dependence on contact-based methods.

In response to these challenges, exploring novel instrumental approaches becomes crucial. These approaches aim to enable the assessment of pulse wave velocity (PWV) with fewer approximations [36]. The goal is to achieve precise evaluations while also considering cost-effectiveness. This pursuit aligns with the ongoing shift towards non-invasive and non-contact solutions in CV monitoring [22]. These advancements are intended to seamlessly integrate into clinical routines for more effective and accurate patient care.

## 3. GMI Transducer

Section 3.1 presents the methods and procedures employed to obtain the characteristic curves of the GMI sensor used in this work. The typical curves of the impedance magnitude and phase of the GMI samples as a function of the magnetic field are analyzed, and the electrical model of the samples is obtained. Section 3.2 describes the electronic circuit developed for the GMI transducer based on the phase characteristics of GMI sensors. Finally, Section 3.3, Section 3.4 and Section 3.5 provide detailed information on the key aspects of the developed transducer, such as sensitivity, linearity, frequency response, noise spectral density, and resolution.

### 3.1. Experimental Characterization of the GMI Sensor

The sensitivity of the GMI pressure transducer is directly related to the sensitivity of the GMI sensor, which is affected by various parameters such as the amplitude, frequency, and DC level of the excitation current; dimensions (length, width, thickness) of the GMI samples; polarization magnetic field (generated by an external source); among others [32]. In order to implement a transducer based on reading the phase characteristics of the GMI sensor element to optimize the transducer sensitivity, it is necessary to define the parameter set responsible for maximizing the sensor’s phase sensitivity (S_phase_), which is given by

(2) $$S_{phase}=\frac{d\theta_{sens}(H)}{dH}$$

where θ_sens_ is the phase of the sensor impedance, and H is the external magnetic field.

The quantitative modeling of the sensitivity dependence on all affecting parameters is still not well-established. Consequently, the search for the optimal point is generally empirical. However, a new optimization technique based on genetic algorithms was proposed in [37] to define the parameter set that maximizes the sensitivity of GMI sensors with different chemical compositions and dimensions (length, thickness, and width). Experimental measurements using this system for a GMI tape sample with a chemical composition of (Co_94_Fe_6_)_72.75_Si_12.25_B_13.25_Cr_1.75_, a length of 3 cm, an average width of 1.5 mm, and a thickness of 45 µm indicated an optimal phase sensitivity (S_phase_) of 10.552°·Oe^−1^, achieved with an excitation current of 40 mA DC level superimposed with a sinusoidal component of 30 mA amplitude and 700 kHz frequency. Thus, these parameters were chosen for the excitation current (i_c_), given by

(3) $$i_{c}=[40+30sin(2\pi (700\times 10^{3})t)]\;mA$$

Although the SI unit for magnetic field is A/m, the work employs oersted (Oe) to facilitate comparative analysis, as Oe is commonly used in the GMI literature for the magnetic field unit. It should be noted that 1 Oe is approximately equivalent to 79.577 A/m.

#### 3.1.1. GMI Sample Characterization System

For the characterization of GMI samples, a Helmholtz coil generates a continuous and uniform magnetic field over the sample volume, with adjustable magnitude through a controllable current source. The Helmholtz coil is a conventionally used structure for generating low-frequency magnetic fields. It consists of two circular coils with identical diameters, separated by a distance equal to the radius of the coils. Each coil has the same number (N) of turns through which a given current (I) flows.

The magnetic field (H) generated at the center of the Helmholtz pair, as a function of the current (I) flowing through its turns, can be calculated using Biot-Savart’s law, given by

(4) $$H=\frac{8\cdot \mu_{0}\cdot N\cdot I}{5\cdot R\cdot \sqrt{5}}$$

where N is the total number of turns in each coil, R is the radius of the coils, and μ_0_ is the magnetic permeability in a vacuum.

The Helmholtz coil used in the experimental characterization has 48 turns in each coil and a radius of 15 cm. Therefore, the magnetic field H, in oersteds, as a function of the current I, in amperes, is approximately given by

(5) H≅2.877⋅I

**Figure 3:**
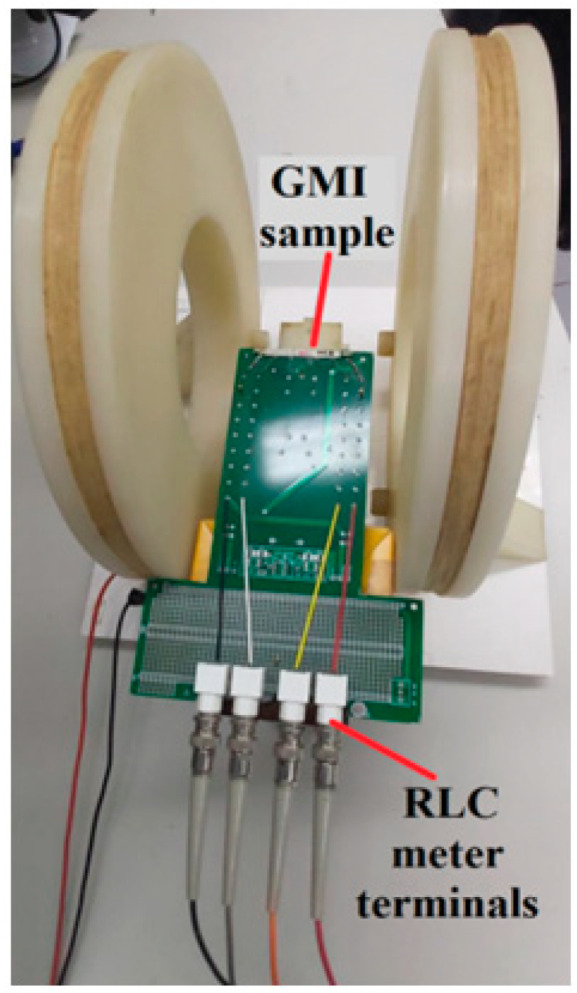
Helmholtz coil with a GMI sample positioned at its centre.

The GMI sensor is placed at the center of the Helmholtz coil and positioned such that the field generated by the coil pair is longitudinal to the length of the sample. In order to minimize the influence of the Earth’s magnetic field in the measurements, the sensor-coil assembly is positioned so that the length of the sensor sample is perpendicular to the direction of the Earth’s field. This adjustment is made with the aid of a compass. Figure 3 shows a photo of the actual structure used in this work.

The characterization system also includes an RLC meter (4285A, Agilent Technologies, Santa Clara, CA, USA) responsible for reading the module and phase of the sample. It should be noted that this meter can also excite the GMI sample with the desired current, allowing for adjustment of the current parameters: DC levels between 0 mA and 100 mA, rms amplitudes between 0 mA and 20 mA, and frequencies between 75 kHz and 30 MHz.

#### 3.1.2. Experimental Characterization

The characterization system described in the previous subsection was employed to experimentally obtain the impedance’s magnitude and phase characteristic curves of the analyzed GMI sensor as a function of the magnetic field. The measurements were performed in order to evaluate hysteresis effects, which often affect the behavior of magnetic sensors. Thus, the measurements started at H_start_ = 0 Oe. From this point, the field was gradually increased up to a maximum value of H_max_ = 2 Oe. Then, the field was reduced to a minimum value of H_min_ = −2 Oe. Finally, the hysteresis cycle was completed by traversing the path from H_min_ to H_start_. The measurements were carried out using a step size of ΔH = 0.01 Oe, and at each step, the corresponding magnitude and phase values of the sample were measured. Following the described procedure, two magnitude and phase values are obtained for each evaluated magnetic field value, except for the extremes of the H_max_ and H_min_ curve, which have only one value, and for H_start_, which has three associated values.

**Figure 4:**
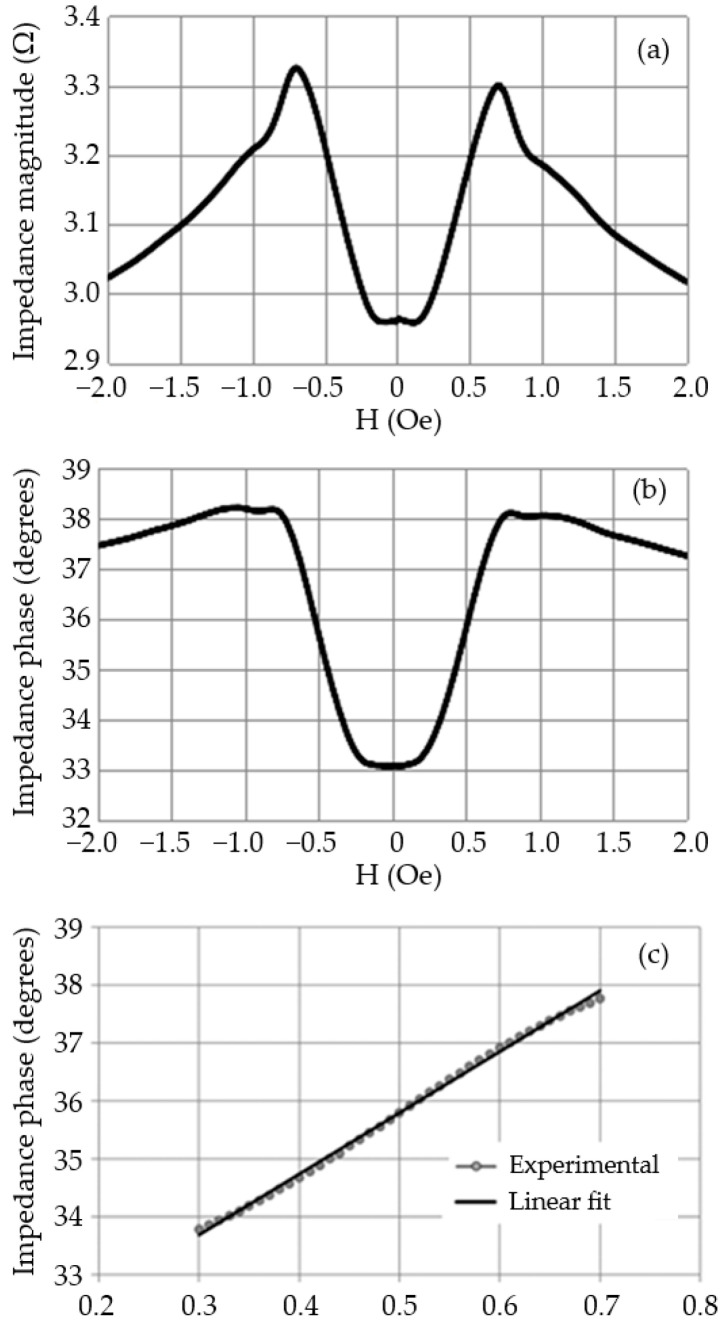
Average curves of (**a**) magnitude and (**b**) phase of the GMI sample with a length of 3 cm, excited by i_C_ = [40 + 30sin(2π(700 × 10^3^)t)] mA. (**c**) Linear Region of the average phase curve of the GMI sample (0.3 Oe ≤ H ≤ 0.7 Oe).

The average curves of the magnitude and phase of the GMI sample’s impedance are shown in Figure 4a,b. These curves are calculated by averaging the point-to-point hysteresis curves.

By analyzing the phase curve obtained in Figure 4b, it can be noticed that the region between 0.3 Oe and 0.7 Oe exhibits high sensitivity and satisfactory linearity. Consequently, it was decided to operate the sensor element within this region to ensure the linearity of the transducer’s response and maximize its sensitivity. It is important to bias the sensor at H = 0.5 Oe (midpoint of the operating region) to maximize symmetrical excursion within this region. Figure 4c provides a more detailed view of the selected operating region.

The coefficient of determination (R^2^) was used to determine how close the data points were to the fitted regression line. Remember that the value of R^2^ ranges from 0 to 1, where a higher R^2^ indicates a better fit of the data to the linear model. The obtained R^2^ value for the linear fit was 0.998, indicating a satisfactorily linear behavior of the phase with respect to the magnetic field. Therefore, it can be observed that the defined linear fitting polynomial adequately models the experimental behavior. Equation (6) presents the fitting polynomial for the interest range (0.3 Oe ≤ H ≤ 0.7 Oe).

(6) $$\theta_{sens}=(10.552{}^{\circ}\cdot Oe^{-1})H+30.521{}^{\circ}$$

#### 3.1.3. GMI Sample Electrical Model

The impedance Z_sens_ of the analyzed GMI samples can be electrically modeled as a resistance R_sens_ in series with an inductance L_sens_, whose values vary depending on the applied external magnetic field [15,38,39], as defined by

(7) $$Z_{sens}(H)=R_{sens}(H)+j\omega L_{sens}(H)$$

where ω is the angular frequency of the sample’s excitation current.

From the magnitude (|Z_sens_|) and phase (θ_sens_) results presented in Figure 4a,b, it is possible to calculate the respective values of resistance R_sens_ and inductance L_sens_ using Equations (8) and (9).

(8) $$R_{sens}(H)=\left|Z_{sens}(H)\right|\cos\theta_{sens}(H)$$

(9) $$L_{sens}(H)=\frac{\left|Z_{sens}(H)\right|sin\theta_{sens}(H)}{\omega }$$

It is worth noting that obtaining the electrical model of the samples is essential for performing computational simulations of the electronic transduction circuit.

### 3.2. Electronic Transduction Circuit

The terminals of the GMI sensor are connected to the electronic transduction circuit described in [17]. This circuit has the functions of electrically powering the GMI sample and reading the phase variation of the sensor element’s impedance, resulting from the field variation ΔH generated by the magnet approaching the sensor sample when a pressure ΔP is applied to the semirigid membrane. Subsequently, this change in the sensor element’s impedance phase is converted into a voltage directly associated with the pressure variation ΔP to which the semirigid membrane was subjected. Note that, strictly speaking, this circuit functions as a magnetic transducer and is an integral part of the pressure-to-electrical voltage transduction chain, which is based on pressure-to-magnetic field transduction and, subsequently, on electrical voltage.

**Figure 5:**
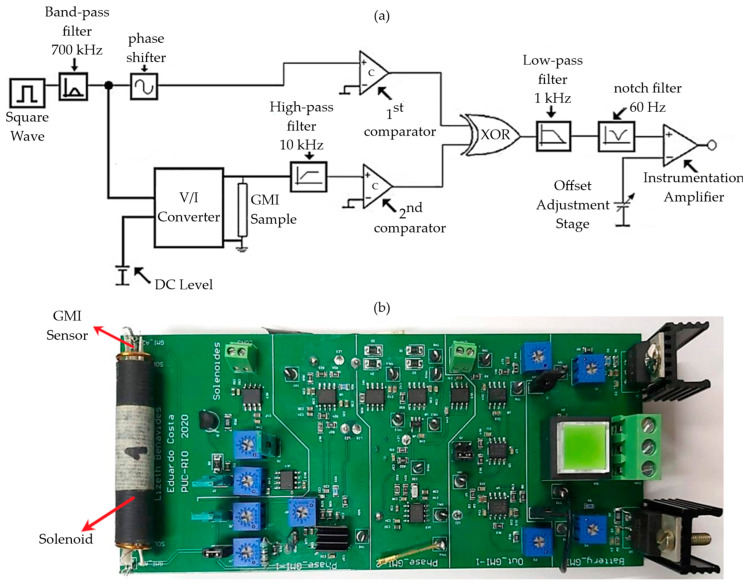
(**a**) Block diagram of the magnetic field-to-voltage electronic transduction circuit and (**b**) Final version of the magnetic field-to-voltage electronic transduction circuit.

The simplified representation of the circuit is shown in Figure 5a through a block diagram.

As indicated in Figure 5a, the transduction circuit begins with an excitation stage composed of an oscillator responsible for generating a square wave with a frequency of 700 kHz, which passes through an 8th-order band-pass filter centered at 700 kHz. Thus, this highly selective filter emphasizes the fundamental component of the square wave (700 kHz) while attenuating the other spectral components. Consequently, the output signal of the filter becomes a 700 kHz sinusoidal wave.

Next, this 700 kHz sinusoidal wave is connected to the input of two blocks: a phase shifter and a voltage-to-current converter (V/I converter), which are adjusted to convert the sinusoidal voltage wave into an alternating current with the same frequency and amplitude, set to 30 mA. Additionally, the V/I converter receives a DC level provided by a voltage regulator and converts it into a continuous current of 40 mA. Finally, these currents are superimposed to excite the GMI sensor with the specified current in Section 3.1, i.e., i_c_ = [40 + 30sin(2π(700 × 10^3^)t)] mA.

In turn, the phase reading stage begins with two comparators configured as null detectors, used to convert the input sinusoidal signals into a square wave with voltage levels compatible with the inputs of the exclusive OR (XOR) gate. The first comparator is connected to a reference sinusoidal signal with a frequency of 700 kHz coming from the output of the phase shifter, whose phase is fixed and independent of the magnetic field. On the other hand, the second comparator receives a signal from the V/I converter where the GMI sensor is connected, which varies its phase according to the magnetic field (H).

It should be noted that the output signal of the V/I converter cannot be directly connected to the input of the second comparator, configured as a null detector, because it never passes through zero volts due to the presence of a DC voltage level. Consequently, in order to remove the DC voltage level, a high-pass filter with a cutoff frequency adjusted to approximately 10 kHz is used. This eliminates the DC level of the signal and allows the passage of the component of interest with a frequency of 700 kHz. This stage is also responsible for amplifying the 700 kHz sinusoidal signal by approximately ten times to provide more suitable voltage levels to the inputs of the second comparator. Therefore, variations in the magnetic field H will induce changes in the phase of the second comparator while the phase of the first comparator remains fixed.

Next, the outputs of the comparators are connected to the inputs of the exclusive OR (XOR) gate, causing it to generate a square wave at its output with an approximately 50% duty cycle and a frequency of 1400 kHz, i.e., twice the frequency of the signals connected to its inputs. For this purpose, the phase shift introduced by the phase shifter is adjusted to ensure that the square waves generated by the two comparators are 90 degrees out of phase with each other for the bias field. It is important to note that this duty cycle is a function of the phase difference between the input waves and will, therefore, vary with the magnetic field H since the phase of the output signal of the second comparator depends on H.

Then, the output waveform of the XOR gate passes through a 4th-order low-pass filter with a cutoff frequency of 1 kHz, which extracts the DC level from the XOR output signal, proportional to the phase difference. In the subsequent steps of the transduction chain, the output signal of the low-pass filter is connected to the input of a notch filter and adjusted to be tuned to 60 Hz, ensuring the attenuation of disturbances coming from the power grid. Finally, the output signal of the notch filter is connected to the input of a low-noise 1/f instrumentation amplifier with a gain adjusted to 50 *V*/*V*. As highlighted in Figure 5a, one of the inputs of the instrumentation amplifier is connected to an offset adjustment stage, which is implemented to ensure that the circuit’s output is zero when the sensor is subjected only to the bias field. Thus, the output of the instrumentation amplifier (circuit output) will be proportional to the phase variations of the GMI sensor element and, consequently, to the applied magnetic field.

The experimental assembly of the circuit described in [17] was done on a breadboard. However, in this work, the decision was made to design and fabricate a printed circuit board since the device configuration is intended for biomedical applications, such as arterial pulse wave measurements and propagation velocity. In this way, the intention is to minimize electronic noise, which can compromise the system’s performance. Additionally, the board was designed to reduce its dimensions, making positioning the device in the areas of interest easier.

The PCB (Printed Circuit Board) development was carried out using Altium Designer 23, a powerful tool for PCB development that allows designing the schematic of the entire circuit and later modeling the printed circuit board. The PCB layout, including component placement, connections, and board shape and dimensions, is generated based on the schematic. Altium software enables adjusting the size of holes, traces, and pads, as well as manual or automatic routing.

In this work, manual routing was chosen for the PCB design due to the complexity of the board, aiming to have more control over all the fine adjustments of the process. Furthermore, considering the characteristics of the biomedical applications of interest, the goal was to reduce the size of the designed board. Thus, the circuit was designed with Surface-Mounted Device (SMD) components, primarily sourced from Texas Instruments (Dallas, TX, USA), Analog Devices (Wilmington, MA, USA), and Linear Technology (Milpitas, CA, USA). Once the PCB design was completed, a Gerber file was generated for board manufacturing, containing all the essential information such as dimensions, traces, holes, vias, and the number of layers, among other details.

To ensure the reliability and quality of the PCB, considering the small size of the SMD components and traces, conventional manual soldering of the components on the board was not performed. Therefore, the fabrication and assembly of the PCB components were carried out by PCB Brasil. Figure 5b shows the final assembly of the manufactured board. The PCB has a length of 138 mm and a width of 60 mm. The GMI sensor is positioned inside the polarization solenoid at the left end of the PCB to facilitate its alignment with the movable magnetic field source (permanent magnet) located on the elastic membrane.

This circuit is an integral part of the pressure-to-electrical voltage transduction chain, based on pressure-to-magnetic field transduction and subsequently on electrical voltage through the described electronic transduction circuit. The electronic transduction circuit was experimentally evaluated to obtain key parameters such as sensitivity, linearity, frequency response, noise spectral density, and resolution. The experimental results of the electronic transduction circuit are presented in detail in the following subsections. In summary, the experimental analysis of the developed electronic transduction circuit indicated an average sensitivity of 19.599 V/Oe, or equivalent to 0.196 mV/nT in the operating region (0.3 Oe ≤ H ≤ 0.7 Oe) and a resolution of 93.105 nT in the passband (0 to 1000 Hz).

### 3.3. Sensitivity and Linearity

**Figure 6:**
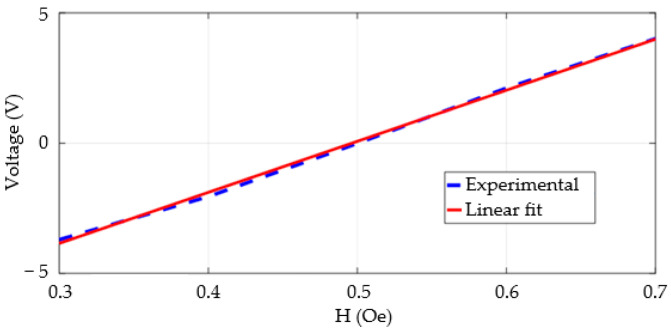
Experimental measurement results of the circuit output voltages for different H values expressed in Oe.

In order to evaluate the sensitivity and linearity of the circuit, the experimental characterization of the magnetometer output voltage as a function of the magnetic field was performed. This procedure consisted of experimentally measuring the circuit output voltages for fields ranging from 0.3 Oe to 0.7 Oe in steps of 0.1 Oe, i.e., within the linear operating range of the GMI sensor used—see Section 3.1. The results of these measurements are shown in Figure 6.

As expected, the results presented in Figure 6 indicate that the circuit output voltage is approximately zero for the bias field (H = 0.5 Oe). It is also possible to observe that, within the proposed operating range, the response of the experimental circuit output voltage as a function of the magnetic field is satisfactorily linear, with a coefficient of determination R^2^ equal to 0.998. Consequently, it is possible to adequately model the behavior of the experimental output voltage using a linear fitting polynomial, given by

(10) $$V_{out}=(19.599\;V\cdot {\mathrm{Oe}}^{-1})\cdot H-9.727\;V$$

Thus, it is found that the average sensitivity of the experimental circuit is 19.599 V/Oe in the operating region.

### 3.4. Frequency Response

In this section, the frequency response of the developed GMI magnetometer was experimentally evaluated to estimate its passband. For this purpose, the dependence of its sensitivity on the frequency of the magnetic field of interest was inspected.

In this setup, the sensor sample was placed in the center of a Helmholtz coil used to generate sinusoidal magnetic fields with known amplitudes and frequencies. To this end, the Helmholtz coil was excited by a low-noise current source (B2962A, Keysight), which can be used as a current or voltage signal generator.

It was chosen to keep the amplitude of the magnetic field fixed at 10 µT, or equivalently 0.1 Oe, in all presented measurements. This amplitude value corresponds to a total excursion of 0.2 Oe within the linear range of the transducer. Thus, it can be considered that the sensitivity remains approximately constant within this range. Any distortions in the output sinusoid of the electronic transduction circuit may be associated with nonlinearities in the response.

**Figure 7:**
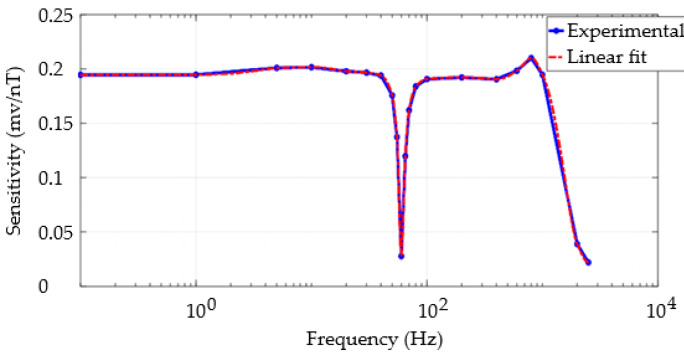
Dependence of the magnetometer sensitivity on the frequency of the excitation magnetic field.

A systematic analysis of the circuit output voltage as a function of the frequency of the excitation magnetic field was performed, with the amplitude kept constant in all tests. Figure 7 presents the behavior of the circuit sensitivity as a function of frequency in hertz. The experimental sensitivity curve in mV/nT was modeled using a fitting polynomial with the MATLAB R2023b curve fitting toolbox. In particular, this toolbox’s interpolant category allows for obtaining point-by-point signal estimates. In this case, the cubic Hermite method, which connects a set of consecutive points and describes them using third-degree polynomials, was used. This tool was employed to obtain the estimated sensitivity curve, Ssens, expressed in mV/nT, as a function of frequency f, expressed in Hz.

By analyzing the behavior of the curve presented in Figure 7, it can be observed that the bandwidth of the developed transducer is approximately 1000 Hz (−3 dB). This result was foreseen since the circuit’s output stage has a low-pass filter with a cutoff frequency set to 1000 Hz. As expected, it is also noticeable that the notch filter effectively rejects the 60 Hz frequency and has minimal impact on neighboring frequencies due to its high selectivity. Additionally, it can be observed that the sensitivity remains satisfactorily constant for frequencies within the passband.

In Figure 7, it can be observed that the fitting curve adequately models the experimental behavior of the sensitivity. This aspect is highly desirable since the fitting polynomial will convert voltage values into magnetic flux density values to obtain the spectral noise density curve expressed in nT·Hz^−1/2^, as will be presented next.

### 3.5. Noise Analysis and Resolution

This subsection presents the experimental evaluation of the noise spectral density of the developed GMI magnetometer. The noise spectral density is a commonly used figure of merit in the characterization of magnetic transducers and is expressed in nT/√Hz.

To obtain the magnetometer’s noise spectral density curve, the sample was subjected only to the bias magnetic field (H = 0.5 Oe), and the output voltage of the transduction circuit was acquired by a data acquisition board (NI USB-6221, National Instruments, Austin, TX, USA). The data acquired by the board is sent to a computer via a USB 2.0 (Universal Serial Bus) connection to be processed by software developed in the LabVIEW environment from National Instruments. LabVIEW provides a VI called “FFT Power Spectrum and PSD” for calculating the noise spectral density.

**Figure 8:**
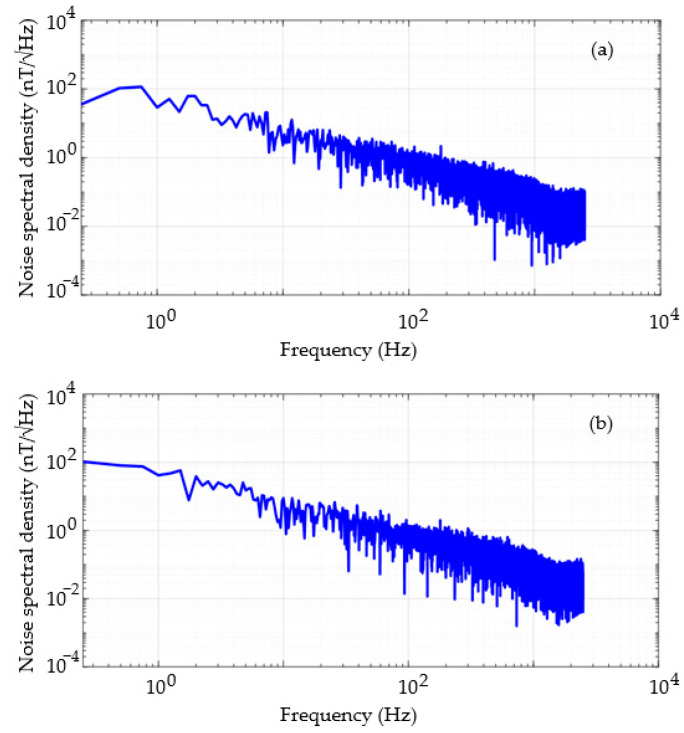
Noise spectral density of the output voltage of the developed magnetometer, expressed in nT/√Hz, using an average of (**a**) 10 and (**b**) 30 samples.

Considering that the magnetometer’s sensitivity is given by the fitting curve presented in Figure 7, it is possible to convert the output voltage into magnetic flux density to obtain the noise spectral density curve expressed in nT/√Hz, as shown in Figure 8. The average of 10 and 30 samples was performed to attenuate the noise levels, and the results are presented in Figure 8a and 8b, respectively.

The curves presented in Figure 8 follow the typical behavior of noise spectral density curves of magnetic transducers, which conventionally exhibit an inverse proportional dependence on frequency power, hence also referred to as 1/*f* noise curves.

From the 1/*f* noise curves, it is possible to estimate the resolution of the developed magnetometer. The noise spectral density curves presented in Figure 8a,b can be approximated by fitting polynomials (g(*f*)). Thus, it is possible to infer the resolution by integrating the square of these polynomials within the frequency range of interest and subsequently calculating the square root of the obtained value, as presented in

(11) $$R=\sqrt{\int_{f1}^{f2}g^{2}(f)df}$$

where *f*_1_ is the lower limit and *f*_2_ is the upper limit of the frequency range of interest.

Based on the information presented in Table 1, the resolution improves with an increase in the number of samples used for averaging. On the other hand, the resolutions in the 0 Hz to 30 Hz range are very close to those obtained for the entire passband. However, it is noted that the resolutions in the 1 Hz to 30 Hz range are substantially better. This behavior was expected since noise levels are significantly higher in the low-frequency region.

## 4. Pulse Wave Measurements

The study was approved by the Research Ethics Review Committee of the Pontifical Catholic University of Rio de Janeiro (045/2020—protocol 83/2020), and the research participant provided informed consent.

Considering the high sensitivity of the GMI magnetic transducer, studies were conducted on the acquisition of pulse wave morphology by using a system composed only of the developed GMI magnetometer and a magnetic marker adhered to the skin surface. To perform this procedure, a small set of magnetized iron filings wrapped in hypoallergenic adhesive tape is positioned on the participant’s skin in a region suitable for measuring the pulse wave of the selected artery. A rotating machine was utilised to grind a fragment of iron into fine particles smaller than one millimetre in diameter, typically ranging from 0.01 to 0.1 mm. The mass of the iron particles was negligible to the touch, so effectively, the total moving mass corresponds to that of the anti-allergic adhesive plaster used to encase them. The static magnetic field detected at a vertical distance of ~2 mm from the marker was approximately 15 µT, providing a sufficient baseline signal-to-noise ratio for detecting dynamic variations during the cardiac cycle. Then, the developed GMI magnetometer is brought close to the area of interest. The pressure variation caused by the arterial pulse wave causes the displacement of the magnetic particles, thereby varying the magnetic field over the GMI sensor. In turn, the field variation alters the impedance phase of the sensor element, which is subsequently converted into a voltage. This procedure can provide some practical advantages, such as not exerting pressure on the patient skin, avoiding a potential risk of reducing the diameter of the arterial wall, and also contributing to better spatial resolution since this parameter is now limited only by the dimension of the magnetic marker, which conventionally has diameters in the order of millimeters or less. This strategy enables access to anatomical regions that are challenging to reach for conventional pressure transducers. However, it should be noted that, without the use of mechanical amplification (Figure 1), for the same pressure variation in the artery, this technique produces much lower variations in the magnetic field over the sensor, making the use of a highly sensitive magnetometer necessary.

**Figure 9:**
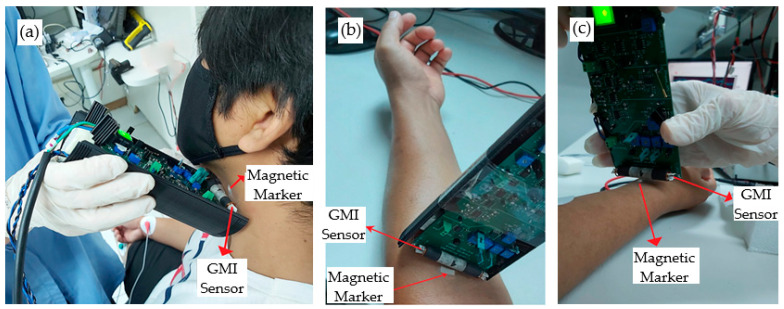
Experimental setup implemented for the direct measurement of the pulse wave, without mechanical amplification, using the magnetometer and magnetic marker positioned at the pulse wave measurement points: (**a**) carotid, (**b**) brachial, and (**c**) radial.

Therefore, considering the high sensitivity of the developed GMI magnetometer, it was sought to employ it in the direct measurement of the pulse wave at different points in the arterial tree. For this purpose, as indicated in Figure 9, the small set of magnetized iron filings wrapped in hypoallergenic adhesive tape was adhered to the skin in the region suitable for pulse wave measurements of the Carotid, Brachial, and Radial arteries. Subsequently, the sensor element of the GMI magnetometer was positioned near each measurement point, adjusting the angle until the acquired signal was stable and of satisfactory amplitude.

**Figure 10:**
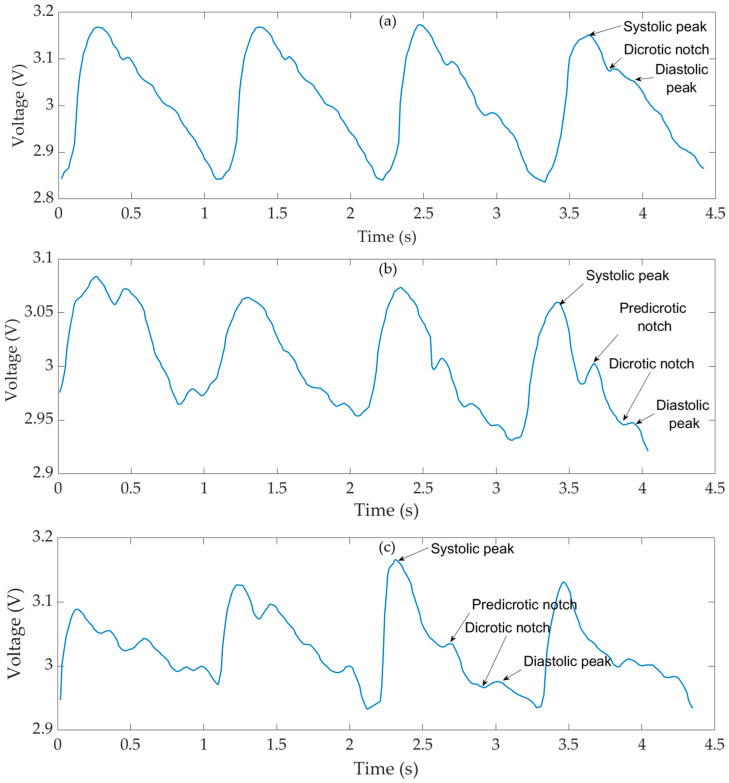
Result of experimental measurements of pulse waves: (**a**) carotid, (**b**) brachial, and (**c**) radial acquired with the magnetometer and magnetic marker adhered to the research participant’s skin.

The GMI magnetometer was manually positioned near the patient’s skin, without applying pressure, over the anatomical regions corresponding to the (a) carotid, (b) brachial, and (c) radial arteries. During measurements, the distance between the GMI sensor and the skin surface was maintained at approximately 2–3 mm. The transducer output signals were acquired by a high-resolution oscilloscope and subjected to digital filtering. The obtained records at the three points in the arterial tree are presented in Figure 10.

The transducer configuration, consisting of a highly sensitive magnetometer and a magnetic marker adhered to the participant’s skin, allowed the recording of pulse waves at three points in the arterial tree: carotid, brachial, and radial. The acquired biosignals have reached satisfactory amplitude levels. As evidenced in Figure 10, the direct measurements with the GMI magnetometer resulted in carotid pulse wave records (Figure 10a) with an amplitude of 0.34 V. In comparison, the brachial and radial pulse waves have values of 0.13 V and 0.19 V (Figure 10b and Figure 10c), respectively.

The recorded pulse waveforms clearly exhibit physiologically relevant features such as the systolic peak, dicrotic notch, and diastolic decay. These landmarks are consistent with the expected morphology of arterial pulse waves as described in the biomedical literature [40].

Although the proposed system captures clear arterial waveforms, minor fluctuations were observed due to skin movement and instability in keeping the sensor in a fixed position, particularly in distal arteries. These effects could be mitigated through improved fixation mechanisms or multi-sensor averaging.

## 5. Estimation of Pulse Wave Velocity (PWV)

This section concerns the estimation of pulse wave velocity (PWV) based on the signals acquired with two highly sensitive GMI magnetometers. In the proposed configuration, a small set of magnetized iron filings wrapped in hypoallergenic adhesive tape is placed on the skin surface in an appropriate region for pulse wave measurement from the selected artery. Then, the high-sensitivity magnetometer is brought close to the filings to capture the signal.

Typically, the arrangement used for PWV measurement is based on the simultaneous measurement of pulse waves at two different positions. PWV is computed by the ratio of the separation distance of the arterial segment and the time it takes for the pulse wave to travel this segment, as explained in Section 2.

In this work, the measurements were taken at two points of the arterial tree: one in the carotid artery and another in the radial artery. Furthermore, it is emphasized that the pulse wave velocity values are affected by the algorithm used to calculate the time delay (*dT*) and the method employed in measuring the distance between the two recording sites (*AB*). The length of the arterial segment AB was estimated by measurements taken with a measuring tape, considering the magnetic marker location as a reference. The distance was measured considering the direct and subtraction methods described in Section 2.

**Figure 11:**
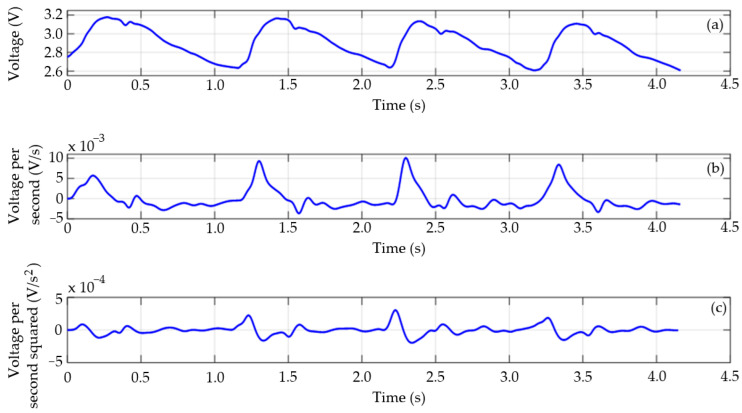
(**a**) Filtered signal, (**b**) first derivative, and (**c**) second derivative of a carotid pulse wave signal acquired with the magnetometer and a magnetic marker adhered to the research participant’s skin.

For the estimation of *dT*, the “foot-to-foot” method commonly used in time delay analysis was implemented. This method employs the values associated with the start instants of the acquired pulse waves at the two measurement points as a reference. These signal instants indicate when an arterial pulse reaches the measurement region. To this end, an algorithm capable of detecting the start point in each recording period was developed. The start point was defined as the maximum value of the second derivative of the signal. The algorithm was developed in MATLAB software, and the derivatives were calculated using the diff function. Figure 11 shows an example of a carotid pulse wave acquired with this system to highlight the performance of the proposed algorithm. It depicts the signal directly acquired at the output of the filtering stage and those obtained after calculating the first and second derivatives.

Since the measurement technique employed for PWV estimation requires the use of two magnetometers, another electronic circuit board with characteristics similar to the one described in Section 3 was developed. The essential parameters of this second prototype magnetometer (sensitivity, linearity, frequency response, spectral noise density, and resolution) are presented in Table 2.

The two GMI magnetometers are brought close to the iron filings attached to the skin over two points in the arterial tree to measure the pulse waves. The pulses are acquired simultaneously to facilitate the measurement of the time interval (*dT*) that a specific pulse wave takes to travel from point *A* to point *B* (Figure 2). Thus, given that the separation distance of the sensors is known, it is possible to calculate PWV as the ratio between the arterial segment separation distance (*AB*) and the time it takes for the pulse wave to traverse this segment (*dT*), as previously presented in Equation (1).

The most intense points of the right carotid and radial pulsation were identified through palpation performed by the examiner and marked on the skin surface at the respective locations to record arterial pulse waves. Subsequently, a small set of magnetized iron filings wrapped in hypoallergenic adhesive tape was placed on the skin surface over the marked locations. For signal acquisition, the research participant remained in the supine position on a bed with the head slightly extended and the right arm relaxed, palm facing upward, in a quiet and comfortable environment.

Two operators acquired the carotid-radial records. The sensor element of the first GMI magnetometer to be built was positioned near the magnetic marker placed on the skin surface at the location of the carotid artery, and simultaneously, the second magnetometer recorded the pulse wave measured in the radial artery (Figure 2).

**Figure 12:**
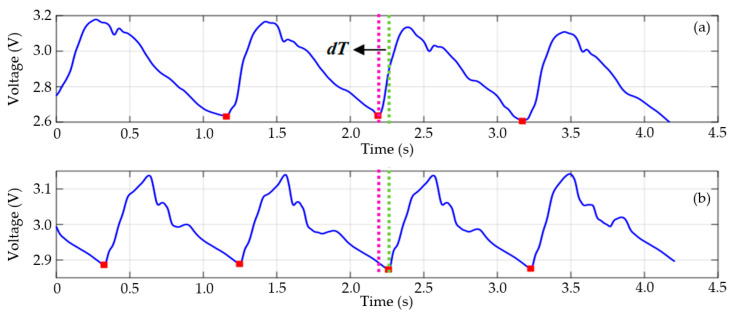
Recordings of the carotid pulse wave in (**a**) and the radial pulse wave in (**b**), acquired synchronously using two GMI magnetometers. The red squares correspond to the starting point of each cycle in the carotid and radial records. The delay time (*dT*) corresponds to the time interval between the pink and green lines.

Figure 12 presents the signals acquired using a high-resolution oscilloscope (HRO 64Zi, Teledyne LeCroy, Chestnut Ridge, NY, USA) and processed using a digital filtering routine implemented in MATLAB. Figure 12a,b display the pulse wave records from the carotid and radial arteries. The signals were measured synchronously by two operators using two GMI magnetometers. The red squares in Figure 12 indicate the start of each wave cycle, as detected by the wave detection algorithm implemented in MATLAB.

Errors in detecting the starting point of the wave can significantly alter the value of the delay time (*dT*) and consequently result in an incorrect estimation of PWV. Therefore, all measurement files were reviewed, and the reference points were thoroughly verified. After determining the starting point of each period, it is possible to estimate the delay time between the records and, finally, the PWV by dividing the separation distance of the arterial segment by the delay time value. It should be noted that PWV is estimated between the two best periods of the acquired wave. For comparison purposes, PWV was calculated using the direct and subtraction methods for distance measurement. These results are presented in Table 3.

As shown in Table 3, the distance value obtained with the subtraction method reveals a difference of 0.094 m when compared to the distance obtained with the direct method. These results corroborate with recent studies that have shown an overestimation of the distance value measured with the direct method, while the distance measured by the subtraction method underestimated the distance value when compared to the actual distance of the branched arterial structure measured by magnetic resonance imaging (MRI) [32].

The observed PWV values ranged from 7.2 to 9.8 m/s, which are in close agreement with normative data for healthy adults, as reported in [32]. The waveform shapes resemble those obtained via applanation tonometry and photoplethysmography, indicating the fidelity of the GMI-based technique.

## 6. Conclusions

Two high-sensitivity magnetometers were developed based on impedance phase readings of GMI samples. Considering that both magnetometers should have similar responses, the authors have selected two GMI samples with similar impedance phase behaviors as a function of the external magnetic field. However, these samples still have a certain level of heterogeneity. More precisely, the GMI sample used in Magnetometer 1 has shown a sensitivity of 10.55 °/Oe, while the GMI sample used in Magnetometer 2 has shown a sensitivity of 8.48 °/Oe. Magnetometer 1 has reached a sensitivity of 19.60 V/Oe, and Magnetometer 2 has a sensitivity of 16.64 V/Oe. Their resolutions (band 1–30 Hz, avg 30) were 62.07 nT and 48.78 nT, respectively.

A method was presented to estimate the pulse wave velocity (PWV) using two GMI magnetometers that perform synchronous acquisition of the pulse wave signals. The results of the experimental measurements for PWV estimation, employing two different methods for measuring the separation distance of the arterial segment, confirmed the possibility of accurate PWV estimation. The estimated pulse wave velocities were within the range of 7 m/s to 10 m/s, which is considered normal according to the literature [32].

Measuring the distance using the subtraction method resulted in a value 15% lower than the result obtained by the direct method, leading to a corresponding variation in PWV values of 15%. This result aligns with recent studies indicating an overestimation by the direct method and an underestimation by the subtraction method compared to MRI-measured distances for branched arterial structures [32]. Low accuracies stemming from distance measurements constitute inherent sources of error in the computation of PWV, where even slight variations can yield substantial deviations in PWV estimates. To ensure meaningful comparisons of PWV estimates among different devices available on the market, it becomes imperative to standardize the distance measurement technique.

Wave pulse measurements using Magneto-Impedance sensors and magnetic markers do not require contact between the transducer probe and a specific area of the skin surface over the artery to be assessed. This approach makes it possible to improve the spatial resolution of conventional measurement systems based on pressure transducers, making it easier to overcome the access limitations caused by anatomical structures close to the arteries whose pulse wave is to be investigated.

This non-invasive, contactless solution avoids applying pressure to the patient’s skin, thereby reducing the risk of decreasing the diameter of the arterial wall, which is typically associated with conventional measurement strategies of arterial wave pulse.

Its ability to detect pulse waves without mechanical compression is beneficial for avoiding applying pressure on sensitive baroreceptors in the artery, such as the carotid sinus, which can trigger hemodynamic reflex responses, particularly in individuals with carotid sinus hypersensitivity (CSH).

Unlike recently developed optical devices for non-contact measurements of arterial pulse waves, the proposed strategy is not affected by ambient light or melanin density on the skin’s surface. The simplicity and non-invasive nature of the proposed system make it suitable for integration into wearable health monitoring devices.

The low-cost manufacturing and operation characteristics of the GMI transducer, along with its safety, non-invasiveness, and high sensitivity, indicate that the developed device meets the requirements advocated by metrology applied to the healthcare sector regarding the development of biomedical instrumentation innovations [21,22].

While the proposed contactless GMI-based pulse wave detection system presents promising results, some limitations must be considered. First, the system requires careful alignment between the GMI sensor and the magnetic marker, as small deviations in distance or angular orientation can affect signal quality. Second, the technique is sensitive to environmental magnetic noise, although this can be mitigated with shielding or differential acquisition strategies. Third, the fixation of the magnetic marker must be consistent to ensure reproducible results, particularly in long-term monitoring or ambulatory scenarios. Additionally, although the present study demonstrates feasibility in healthy individuals, further investigation is required in patients with cardiovascular disorders, where arterial wall dynamics may differ.

Future developments will focus on developing an articulated positioning system that securely attaches the magnetic detection probes in specific locations, based on anatomical context, allowing for accurate measurement of arterial pulse waveforms. In addition, it is intended to conduct a systematic study to optimise the materials’ mass and magnetic flux density for the enclosed iron filings, ensuring they generate an appropriate weak magnetic field source. The studies will aim at further characterising the device’s performance through expanded experimental studies, including measures of clinical disorders, in addition to the current recordings from healthy individuals.

## Figures and Tables

**Table 1:** Resolution of the developed magnetometer.

Average	Resolution (nT) (0–1000 Hz)	Resolution (nT) @ 1 Hz	Resolution (nT) (0–30 Hz)	Resolution (nT) (1–30 Hz)
10	106.712	29.01	105.525	68.952
30	93.105	41.55	91.829	62.067

**Table 2:** Key parameters of the second developed GMI magnetometer.

Magnetometer 2	
Average GMI sample sensitivity (°/Oe)	8.48
Average sensitivity (V/Oe)	16.64
Bandwidth (Hz)	1000
Resolution (band 0–1000 Hz, avg 10) [nT]	74.46
Resolution (band 0–1000 Hz, avg 30) [nT]	64.66
Resolution (1 Hz, avg 10) [nT]	33.89
Resolution (1 Hz, avg 30) [nT]	12.05
Resolution (band 0–30 Hz, avg 10) [nT]	72.31
Resolution (band 0–30 Hz, avg 30) [nT]	63.98
Resolution (band 1–30 Hz, avg 10) [nT]	35.39
Resolution (band 1–30 Hz, avg 30) [nT]	48.78

**Table 3:** PWV estimation based on synchronous measurement of two pulse wave signals.

Distance Measurement Method	Delay Time (s)	Distance (m)	PWV (m/s)
Direct	0.071	0.615	8.662
Subtraction		0.521	7.338

## Data Availability

The raw data supporting the conclusions of this article will be made available by the authors on request.

## References

[1] World Health Organization (2011). Global Status Report on Noncommunicable Diseases 2010.

[2] Salvi P. (2017). Pulse Waves: How Vascular Hemodynamics Affects Blood Pressure.

[3] Mattace-Raso F.U.S., Hofman A., Verwoert G.C., Witteman J.C.M., Wilkinson I., Cockcroft J., McEniery C., Yasmin, Laurent S., Boutouyrie P. (2010). Determinants of pulse wave velocity in healthy people and in the presence of cardiovascular risk factors: ‘Establishing normal and reference values’. Eur. Heart J..

[4] Williams B., Mancia G., Spiering W., Agabiti Rosei E., Azizi M., Burnier M., Clem-ent D.L., Coca A., de Simone G., Dominiczak A. (2018). 2018 ESC/ESH Guidelines for the management of arterial hypertension. Eur. Heart J..

[5] Kim H.L., Kim S.H. (2019). Pulse Wave Velocity in Atherosclerosis. Front. Cardiovasc. Med..

[6] Castelli R., Gidaro A., Casu G., Merella P., Profili N.I., Donadoni M., Maioli M., Delitala A.P. (2023). Aging of the Arterial System. Int. J. Mol. Sci..

[7] Woolam G.L., Schnur P.L., Vallbona C., Hoff H.E. (1962). The pulse wave velocity as an early indicator of atherosclerosis in diabetic subjects. Circulation.

[8] Kis É., Cseprekál O., Horváth Z., Katona G., Fekete B.C., Hrapka E., Szabó A., Szabó A.J., Fekete A., Reusz G.S. (2008). Pulse wave velocity in end-stage renal disease: Influence of age and body dimensions. Pediatr. Res..

[9] Yang H.-H., Chen Y.-C., Ho C.-C., Hsu B.-G. (2024). Serum Phenylacetylglutamine among Potential Risk Factors for Arterial Stiffness Measuring by Carotid–Femoral Pulse Wave Velocity in Patients with Kidney Transplantation. Toxins.

[10] Sharma R.K., Kamble S.H., Krishnan S., Liu I.-C., Gumz M.L., Gomes J., To B., Li S., Mohandas R. (2023). Involvement of lysyl oxidase in the pathogenesis of arterial stiffness in chronic kidney disease. Am. J. Physiol. Renal Physiol..

[11] Lever-Megina C.G., Cavero-Redondo I., Saz-Lara A., Martínez-Vizcaíno V., Álvarez-Bueno C. (2025). Association between pulse wave velocity and cerebral microbleeds: A systematic review and meta-analysis. Hypertens. Res..

[12] Wang H., Wang L., Sun N., Yao Y., Hao L., Xu L., Greenwald S.E. (2020). Quantitative Comparison of the Performance of Piezoresistive, Piezoelectric, Acceleration, and Optical Pulse Wave Sensors. Front. Physiol..

[13] Zieff G., Stone K., Paterson C., Fryer S., Diana J., Blackwell J., Meyer M.L., Stoner L. (2023). Pulse-wave velocity assessments derived from a simple photoplethysmography device: Agreement with a referent device. Front. Cardiovasc. Med..

[14] Silva E.C., Gusmão L.A.P., Barbosa C.R.H., Monteiro E.C. (2009). Magnetic field transducers based on the phase characteristics of GMI sensors and aimed at biomedical applications. Proceedings of the 13th International Conference on Biomedical Engineering IFMBE.

[15] Phan M.H., Peng H.X. (2008). Giant magnetoimpedance materials: Fundamentals and applications. Prog. Mater. Sci..

[16] Costa Silva E., Gusmão L.A.P., Hall Barbosa C.R., Costa Monteiro E., Machado F.L.A. (2011). High sensitivity giant magnetoimpedance (GMI) magnetic transducer: Magnitude versus phase sensing. Meas. Sci. Technol..

[17] Cabrera L., Costa Silva E., Costa Monteiro E., Barbosa C.R.H. (2018). High Sensitivity Pressure Transducer based on the phase characteristics of GMI magnetic sensors. Meas. Sci. Technol..

[18] Cabrera L.S.B., Costa Silva E., Costa Monteiro E. Pressure transducer based on the phase characteristics of GMI effect for measuring the arterial pulse wave. Proceedings of the XXII World Congress of the International Measurement Confederation (IMEKO).

[19] Hill R.D., Smith R., Walker H.K., Hall W.D., Hurst J.W. (1990). Examination of the extremities: Pulses, bruits, and phlebitis. Clinical Methods: The History, Physical, and Laboratory Examinations.

[20] Drzewiecki G.M., Melbin J., Noordergraaf A. (1983). Arterial tonometry: Review and analysis. J. Biomech..

[21] Costa Monteiro E., Leon L.F. (2015). Metrological Reliability of Medical Devices. J. Phys. Conf. Ser..

[22] Costa Monteiro E., Summers R. (2022). Metrological requirements for biomedical device assessment and their ethical implications. Meas. Sens..

[23] Bramwell J.C., Hill A.V. (1922). Velocity of transmission of the pulse-wave and elasticity of arteries. Lancet.

[24] Salvi P. (2012). Pulse Waves: How Vascular Hemodynamics Affects Blood Pressure.

[25] Salvi P., Magnani E., Valbusa F., Agnoletti D., Alecu C., Joly L., Benetos A. (2008). Comparative study of methodologies for pulse wave velocity estimation. J. Hum. Hypertens..

[26] Ribeiro F.A., Thoen R.H., Köhler I., Danzmann L.C., Torres M.A.R. (2012). Síndrome metabólica: Complacência arterial e a velocidade de onda de pulso. Rev. Amrigs..

[27] Mitchell G.F. (2009). Arterial stiffness and wave reflection: Biomarkers of cardiovascular risk. Artery Res..

[28] Elias M.F., Robbins M.A., Budge M.M., Abhayaratna W.P., Dore G.A., Elias P.K. (2009). Arterial pulse wave velocity and cognition with advancing age. Hypertension.

[29] Jannasz I., Sondej T., Targowski T., Mańczak M., Obiała K., Dobrowolski A.P., Olszewski R. (2023). Relationship between the Central and Regional Pulse Wave Velocity in the Assessment of Arterial Stiffness Depending on Gender in the Geriatric Population. Sensors.

[30] Asmar R., Benetos A., Topouchian J., Laurent P., Pannier B., Brisac A.M., Target R., Levy B.I. (1995). Assessment of arterial distensibility by automatic pulse wave velocity measurement: Validation and clinical application studies. Hypertension.

[31] Wilkinson I.B., Fuchs S.A., Jansen I.M., Spratt J.C., Murray G.D., Cockcroft J.R., Webb D.J. (1998). Reproducibility of pulse wave velocity and augmentation index measured by pulse wave analysis. J. Hypertens..

[32] Van Bortel L.M., Laurent S., Boutouyrie P., Chowienczyk P., Cruickshank J.K., De Backer T., Filipovsky J., Huybrechts S., Mattace-Raso F.U.S., Protogerou A.D. (2012). Expert consensus document on the measurement of aortic stiffness in daily practice using carotid-femoral pulse wave velocity. J. Hypertens..

[33] Townsend R.R. (2016). Arterial Stiffness: Recommendations and Standardization. Pulse.

[34] Horváth I.G., Németh Á., Lenkey Z., Alessandri N., Tufano F., Kis P., Gaszner B., Cziráki A. (2010). Invasive validation of a new oscillometric device (Arteriograph) for measuring augmentation index, central blood pressure and aortic pulse wave velocity. J. Hypertens..

[35] De Melis M., Morbiducci U., Scalise L., Tomasini E.P., Delbeke D., Baets R., Van Bortel L.M., Segers P. (2008). A preliminary study for the evaluation of large artery stiffness: A non contact approach. Artery Res..

[36] Pereira T., Correia C., Cardoso J. (2015). Novel Methods for Pulse Wave Velocity Measurement. Med. Biol. Eng..

[37] Benavides Mora J.D., Costa da Silva E. (2018). Development of an automated system based on the concept of evolutionary hardware to determine the optimal operating point of GMI sensors. J. Phys. Conf. Ser..

[38] Zheng G.T., Liu Z.H., Jiang D.G. (2010). GMI effect of FeCoSiB amorphous ribbon. Adv. Mater. Res..

[39] Mohri K., Kohzawa T., Kawashima K., Yoshida H., Panina L.V. (1992). Magneto-Inductive effect (MI effect) in amorphous wires. IEEE Trans. Magn..

[40] Yan J., Wang Z., Guo R., Yan H., Wang Y., Qiu W. (2025). Blood pressure prediction based on multi-sensor information fusion of electrocardiogram, photoplethysmography, and pressure pulse waveform. Measurement.

